# Circulating microRNAs as diagnostic biomarkers for ischemic stroke: evidence from comprehensive analysis and real-world validation

**DOI:** 10.7150/ijms.83963

**Published:** 2023-06-04

**Authors:** Yang Wang, Xianwei Su, Geoffrey Ho Duen Leung, Bohua Ren, Qiang Zhang, Zhiqiang Xiong, Jingye Zhou, Ling Yang, Gang Lu, Wai-Yee Chan, Lijie Ren

**Affiliations:** 1 Department of Neurology, The First Affiliated Hospital of Shenzhen University, Shenzhen Second People's Hospital, Shenzhen 518000, China.; 2 Research and Development Unit, Shenzhen GenDo Medical Technology Co., Ltd., Dapeng, Shenzhen 518000, China.; 3 SDIVF R&D Centre, 209,12W, HKSTP, Shatin, Hong Kong, China.; 4 Department of Epidemiology and Biostatistics, School of Public Health, Guangdong Medical University, Dongguan 523808, China.; 5 Faculty of Education, Health and Wellbeing, University of Wolverhampton, Wolverhampton WV1 1QU, UK.; 6 CUHK-SDU Joint Laboratory on Reproductive Genetics, School of Biomedical Sciences, The Chinese University of Hong Kong, Hong Kong SAR, China.

**Keywords:** circulating microRNAs, ischemic stroke, biomarkers, comprehensive analysis, real-world validation

## Abstract

Ischemic stroke (IS) is the majority of strokes which remain the second leading cause of deaths in the last two decades. Circulating microRNAs (miRNAs) have been suggested as potential diagnostic and therapeutic tools for IS by previous studies analyzing their differential expression. However, inconclusive and controversial conclusions of these results have to be addressed. In this study, comprehensive analysis and real-world validation were performed to assess the associations between circulating miRNAs and IS. 29 studies with 112 miRNAs were extracted after manual selection and filtering, 12 differentially expressed miRNAs were obtained from our results of meta-analysis. These miRNAs were evaluated in 20 IS patients, compared to 20 healthy subjects. 4 miRNAs (hsa-let-7e-5p, hsa-miR-124-3p, hsa-miR-17-5p, hsa-miR-185-5p) exhibited the significant expression level in IS patient plasma samples. Pathway and biological process enrichment analysis for the target genes of the 4 validated miRNAs identified cellular senescence and neuroinflammation as key post-IS response pathways. The results of our analyses closely correlated with the pathogenesis and implicated pathways observed in IS subjects suggested by the literature, which may provide aid in the development of circulating diagnostic or therapeutic targets for IS patients.

## Introduction

Stroke has been ranked as the second leading cause of death globally in the last two decades 1. The majority of strokes are ischemic stroke (IS) which are caused by the blockage of blood supply to the brain due to embolism or other vascular diseases such as atherosclerosis, leading to the lack of oxygen and nutrients supply and brain damage. Current diagnosis for IS is mainly based on the use of computed tomography scan or magnetic resonance imaging after the patient is hospitalized 2. In the past decade, studies have emerged suggesting other potential diagnostic approaches for different diseases including the dysregulation of microRNAs (miRNAs) and circular RNAs 3. miRNAs have become one of the most promising types of biomarkers among these approaches and were extensively studied for their potential use in cancers, nervous system disorders and cardiovascular diseases 4. miRNAs are a class of non-coding, small RNAs composed of around 20-25 nucleotides. They function to regulate the degradation of mRNAs and transcription and translation of their target genes 5, 6. Multiple sources for extracting miRNAs have been suggested, such as plasma, serum, peripheral blood mononuclear cells (PBMCs), cerebrospinal fluid (CSF), saliva, and urine. Circulating miRNAs have been the focus among them due to its ease to detect, high accuracy, specificity and stability 7.

The understanding of the role of miRNAs in stroke patients has been improved in the recent years. Studies have shown that the expression levels of miRNAs were associated with the prognosis of stroke 8, 9. Differentially expressed miRNAs in the circulation or CSF of stroke patients were suggested, raising the potential of using these miRNAs as early diagnostic biomarkers or treatments 10, 11. Studies have also shown the value of particular circulating miRNAs as the predictive tools for stroke risk by the combination of multiple clinical risk factors such as age, sex, smoking status, blood pressure and body mass index. Some of these miRNAs include miR-6124, miR-5196-5p, miR-4292, of which the expression levels in serum were associated with the risk of stroke 12, 13. Previous studies have shown the differential expression levels of many other miRNAs (miR-9, miR-29b, miR124 and miR-125b) and suggested their potential clinical value 14. Despite this, consistency and reliability of the results should be addressed in terms of the differences in number of sample sizes, subjects and miRNA profiling methods. Therefore, we assess the existing studies for circulating miRNAs in stroke patients by performing meta-analysis and experimental validation.

Early prediction and diagnosis for IS is of vital importance to reduce the risk of permanent brain damage due to the delay in prevention and treatments. This study identified 12 miRNAs by meta-analysis as potential circulating biomarkers for IS. 4 miRNAs (hsa-let-7e-5p, hsa-miR-124-3p, hsa-miR-17-5p, hsa-miR-185-5p) exhibited the significant expression level in IS subjects compared to the healthy subjects. The target genes of these miRNAs were closely related to the cellular senescence and neuroinflammation pathways implicated in ischemia. This study will provide a direction for future studies in developing diagnostic and therapeutic targets for IS patients.

## Materials and methods

### Data Collection

Studies were collected on PubMed from 1^st^ January 1994 until 31st December 2022. Studies were searched on PubMed using the following search terms: ("stroke") AND ("Blood" OR "Serum" OR "Plasma" OR "Circulat*" OR "Peripheral" OR "PBMC") AND ("miRNA" OR "microRNA" OR "miR"). The collected studies' title, abstract and full texts were then screened and selected according to the eligibility criteria. Information of each relevant study was recorded in a standardised form including the PubMed ID, first author, publication year, region, specimen(s), miRNA profiling method(s), number of cases and controls, dysregulated miRNAs, their dysregulation states, and p-values.

### Eligibility Criteria

Studies were included in the present meta-analysis if they (1) were primary studies; (2) were case-controlled studies; (3) performed profiling of human miRNAs in the circulation of IS patients; (4) reported the type(s) of specimens used; (5) reported the sample sizes of case and control groups; (6) reported the miRNA profiling methods; (7) reported the statistical significance of each miRNA and their dysregulation states; (8) did not perform irrelevant comparisons (e.g., treatment-naïve vs treatment group); (9) were written in English. Studies that were excluded if they (1) were review articles; (2) did not contain case and control (stroke and healthy) samples; (3) performed profiling of miRNAs in animal models or cell lines other than human blood, plasma or serum; (4) did not report the type(s) of specimens used; (5) did not report the sample sizes of case and control groups; (6) did not report the miRNA profiling methods; (7) did not report the statistical significance of each miRNA and their dysregulation states; (8) performed comparisons other than stroke vs healthy controls; (9) written in languages other than English.

### Meta-analysis to identify differentially expressed miRNAs

Meta-analysis was performed for each miRNA that was differentially expressed in IS patients in more than one study. The names of the miRNAs were standardized by the R package *miRNAmeConverter* (Version 1.10.0) 15 according to the miRBase database (Version 22.0) 16. R package *metafor* (Version 3.0.2) was used to calculate the effect sizes for each qualified miRNA in each study (*θ_i_*) independently as log Odds Ratios (logORs) using a random-effects model 17. The logOR for the *i*th study was calculated by:



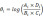



where in a 2x2 table, ***A_i_***, ***B_i_***, ***C_i_*** and ***D_i_*** represent the number of up-regulation events in the disease group, down-regulation events in the disease group, up-regulation events in the control group, and down-regulation events in the control group, respectively. For this meta-analysis, since there were no differentially expressed miRNAs in the control group, ***C_i_*** represents the number of controls and ***D_i_*** would be zero. 0.5 was added to all zero values in the equation. Other outcomes include p-values, tau square (τ^2^), *I*^2^ and the sample variance (*v_i_*). τ^2^ and *I*^2^ were used to estimate the heterogeneity of each miRNA. The weight of the *i*th study (*W_i_*) was calculated by:



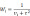



The overall effect size for a miRNA in the associated studies was then calculated by:



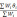



The result was considered significant if the p-value was lower than 0.05. The miRNA was considered up-regulated or down-regulated if the overall effect size was greater or smaller than 0, respectively.

### Tissue-specific expression analysis of 12 miRNAs

miRNAs identified from meta-analysis were subjected to tissue enrichment analysis using data obtained from Human miRNA tissue atlas 18. Quantile-normalized data was used to visualize the expression levels of each miRNA in a total of 31 tissues. Expression levels of multiple samples obtained from the same tissue were grouped into their average values. Relative expression levels of each miRNA across the tissues were calculated as z-scores for visualization.

### RNA extraction from plasma

Human blood plasma was prepared from peripheral blood as described previously 19. Briefly, at 24 hour following stroke onset, blood samples were obtained using EDTA tubes using standard procedures. The samples were placed on ice immediately and centrifuged at 1000 g for 15 minutes at 4°C. Total RNA was extracted from 200 μl plasma using the Universal Extraction Kit following the manufacturer's instructions (GeneDotech, #GD-101). Total RNA was eluted by adding 15 μl of nuclease-free water and stored at -80^o^C. 20 IS patients and 20 healthy subjects were enrolled for this study. The ischemic patients recruited were defined by an acute focal neurological deficit in combination with a diffusion weighted imaging-positive lesion on magnetic resonance imaging or a new lesion on a delayed CT scan. The collection time point was at 24 hour after stroke onset. The collection and use of specimens in this experiment were all signed and confirmed by patients and healthy subjects. The study design was approved by the appropriate ethics review board of The Second People's Hospital of Shenzhen (No. 20200601022-FS01). The consent form was approved by the Medical Ethics Committee of The Second People's Hospital of Shenzhen.

### cDNA synthesis and real time PCR

5 μl eluted RNA was reverse transcribed in 20 μl reactions according to manufacturer's instructions (GeneDotech, #GD-102). Briefly, 5 μl of RNA in a final volume of 20 μl including transcription mastermix was incubated at 42°C for 1 hour followed by enzyme inactivation at 95°C for 5 minutes. The cDNA was diluted and assayed in 10 μl PCR reactions according to the instruction for the PCR master mix (Probe) (GeneDotech, #GD-105). Quantitation of miRNAs was carried out using Probe based Real-Time PCR. The amplification was performed in StepOne plus Detection System (Applied Biosystems) in 96 well plates, each sample is performed in triplicate. The amplification curves were analyzed using the ABI SDS software, both for determination of Ct. The gene expression levels of selected miRNAs are presented as ΔCt relative to the mean Ct values of the external references including cel-miR-39, 54 and 238. Fold change was calculated relative to that of healthy individuals' group. The raw average Ct values of measured miRNAs and external references were displayed in supplementary Table S1.

### Biological Significance

Target genes for each miRNA that was identified from the meta-analyses were retrieved by the R package *multiMiR* (Version 1.4.0) 20. Only the validated miRNA-target gene interactions obtained from mirTarBase were included. Pathway enrichment analysis for these target genes was performed by R packages *ReactomePA* (Version 1.26.0) and *clusterProfiler* (Version 3.10.1) based on Reactome and Kyoto Encyclopedia of Genes and Genomes (KEGG), respectively 21, 22. Gene Ontology (GO) analysis was performed to analyze the enrichment of biological processes. A pathway or biological process was considered significantly enriched if its associated False Discovery Rate (FDR)-adjusted *p*-value was less than 0.05. Ingenuity Pathway Analysis (IPA) was used to further study the miRNA-mRNA interactions and their associated pathways using its microRNA Target Filter module 23. The interactions and their associated pathways were further filtered. Only those with strong confidence (experimentally observed), implicated in cardiovascular disease, neuroinflammatory response or neurological disease, and human interactions were selected. Subsequent miRNA-mRNA interactions network was plotted using PathDesigner included in IPA software.

### Receiver operating characteristics analysis

Receiver operating characteristics (ROC) curve analysis was performed using plasma samples collected from 20 IS patients and 20 healthy individuals from the hospital. Logistic model was built on the normalized expression levels for each miRNA and disease group. R package “pROC” was used to calculate AUC values for each model and visualise the ROC curves.

### Statistical analysis

GraphPad Prism 8.2 and SPSS 19.0 statistical packages were used for statistical analysis, and the Student's t test (two-tailed) was used in qRT-PCR analysis between two groups of data sets. P-value <0.05 was considered statistically significant.

## Results

### Included literatures

**Figure 1:**
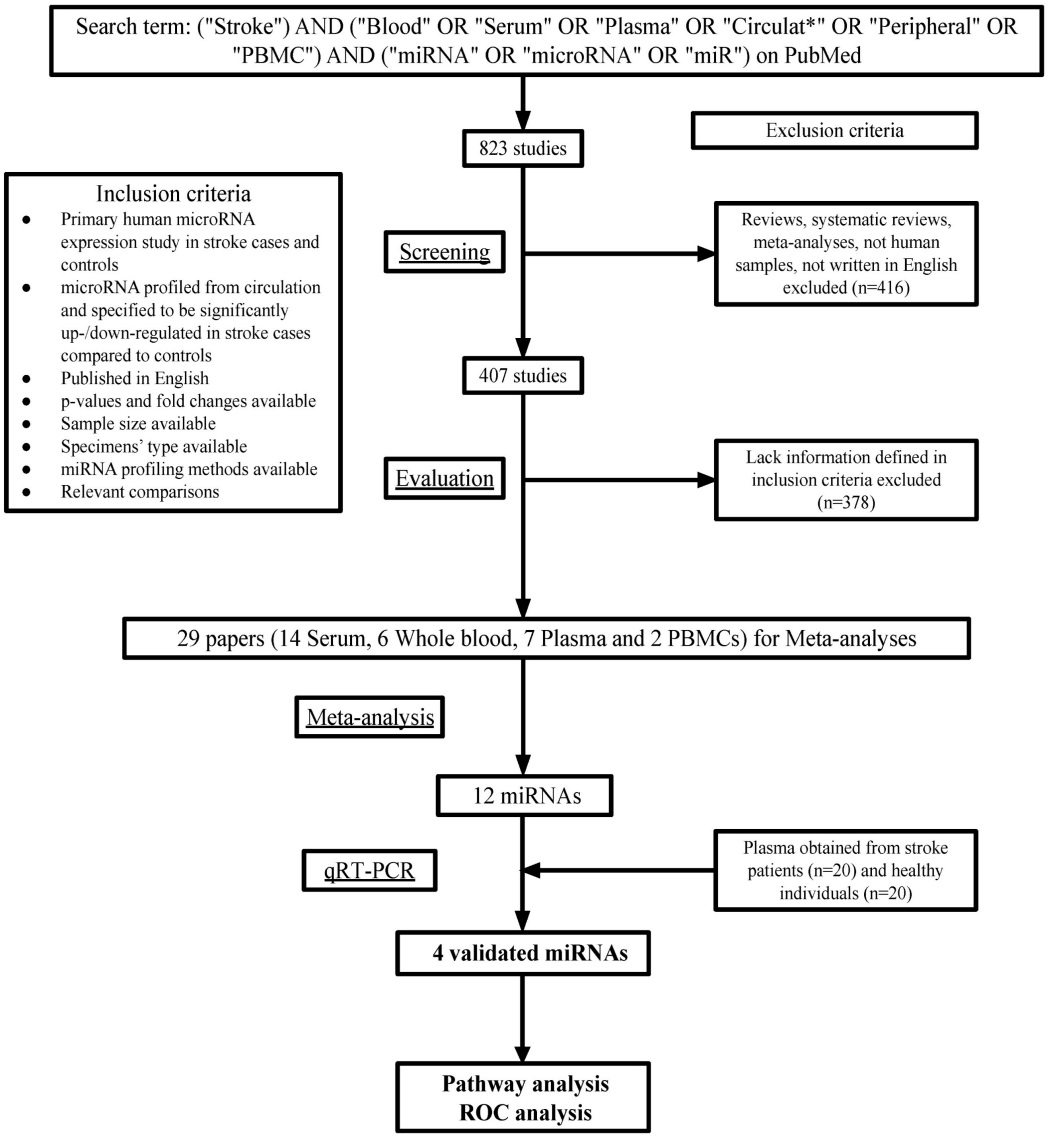
Workflow of meta-analysis and real-world validation stage.

A total of 823 articles were found on PMC using the search terms. After excluding the studies that did not match the eligibility criteria, 29 studies were left (Figure 1). Among these studies, 14 measured the levels of miRNAs in patients' serum, 7 in plasma, 6 in whole blood and 2 in peripheral blood mononuclear cells (Table 1).

### Differential expressed miRNAs from meta-analysis

One hundred and twelve unique miRNAs were included initially by these studies. After standardization, a list of 96 unique miRNAs was generated for this meta-analysis (Table 1). Twenty-three of these miRNAs were suggested by more than 1 study, of which 15 were qualified for performing meta-analysis as their differential expression states were derived from the same blood elements (Table 2). Twelve miRNAs were identified as significantly dysregulated by the meta-analysis with *p* < 0.05. Eight of them were upregulated (hsa-let-7e-5p, hsa-miR-17-5p, hsa-miR-185-5p, hsa-miR-218-5p, hsa-miR-222-3p, hsa-miR-451a, hsa-miR-487b-3p, hsa-miR-9-5p) while 4 were downregulated (hsa-miR-124-3p, hsa-miR-126-3p, hsa-miR-130a-3p, hsa-miR-221-3p).

### Tissue-specific expression levels of 12 miRNAs

**Figure 2:**
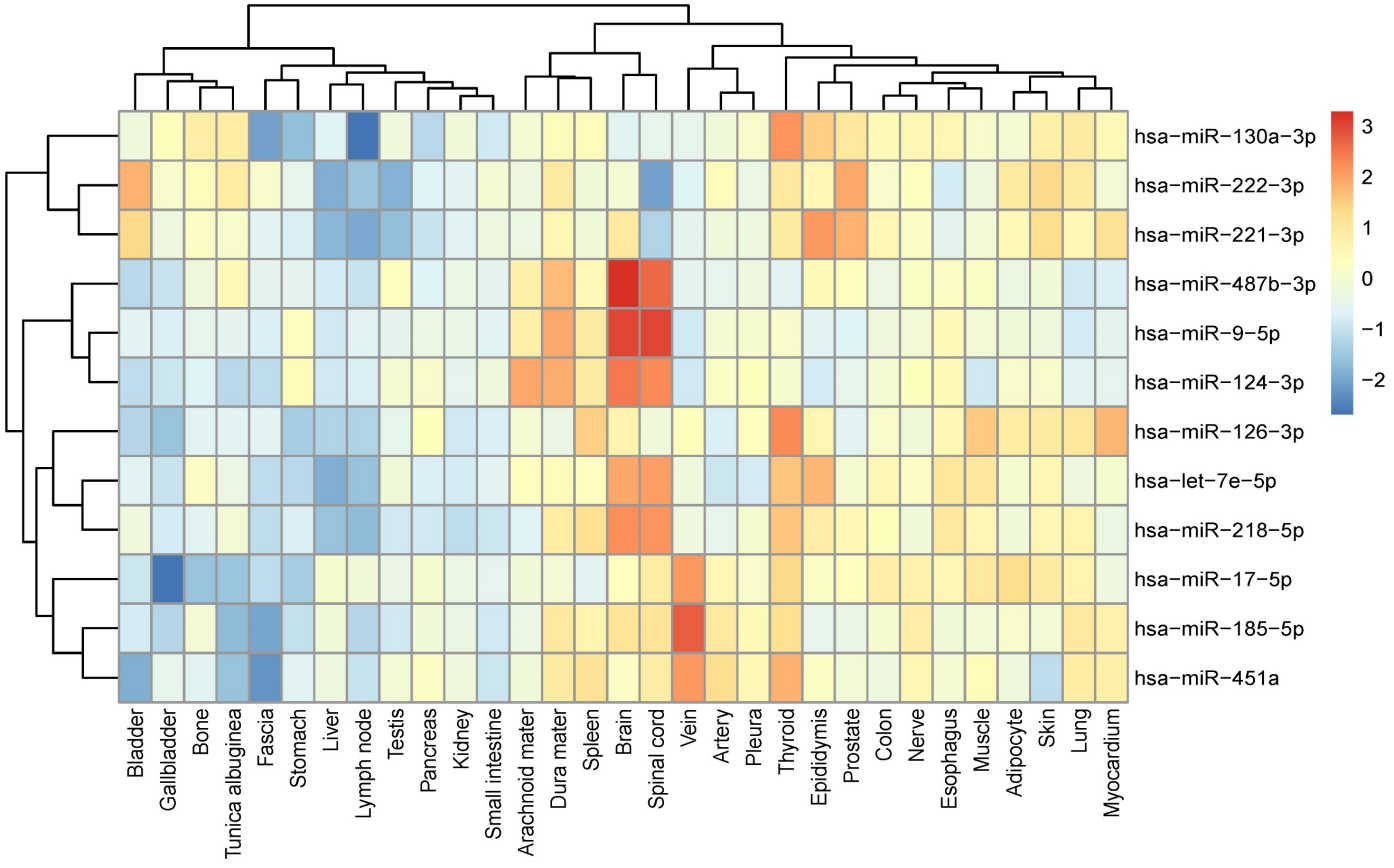
Tissue-specific expression of 12 miRNAs. Relative expression of each validated miRNA in each tissue was plotted using z-scores.

To investigate the relationship between tissue specificity and IS, the expression levels of 12 identified miRNAs in multiple tissues were studied. hsa-miR-487b-3p, hsa-miR-9-5p and hsa-miR-124-3p are specifically expressed in the Central Nervous System (CNS) tissues including arachnoid mater, brain, dura mater and spinal cord, while hsa-let-7e-5p and hsa-miR-218-5p are also highly expressed in the CNS tissues. hsa-miR-17-5p, hsa-miR-185-5p and hsa-miR-451a, on the other hand, are found to be highly expressed in veins (Figure 2).

### Validation of miRNAs in plasma of IS patients using qRT-PCR

**Figure 3:**
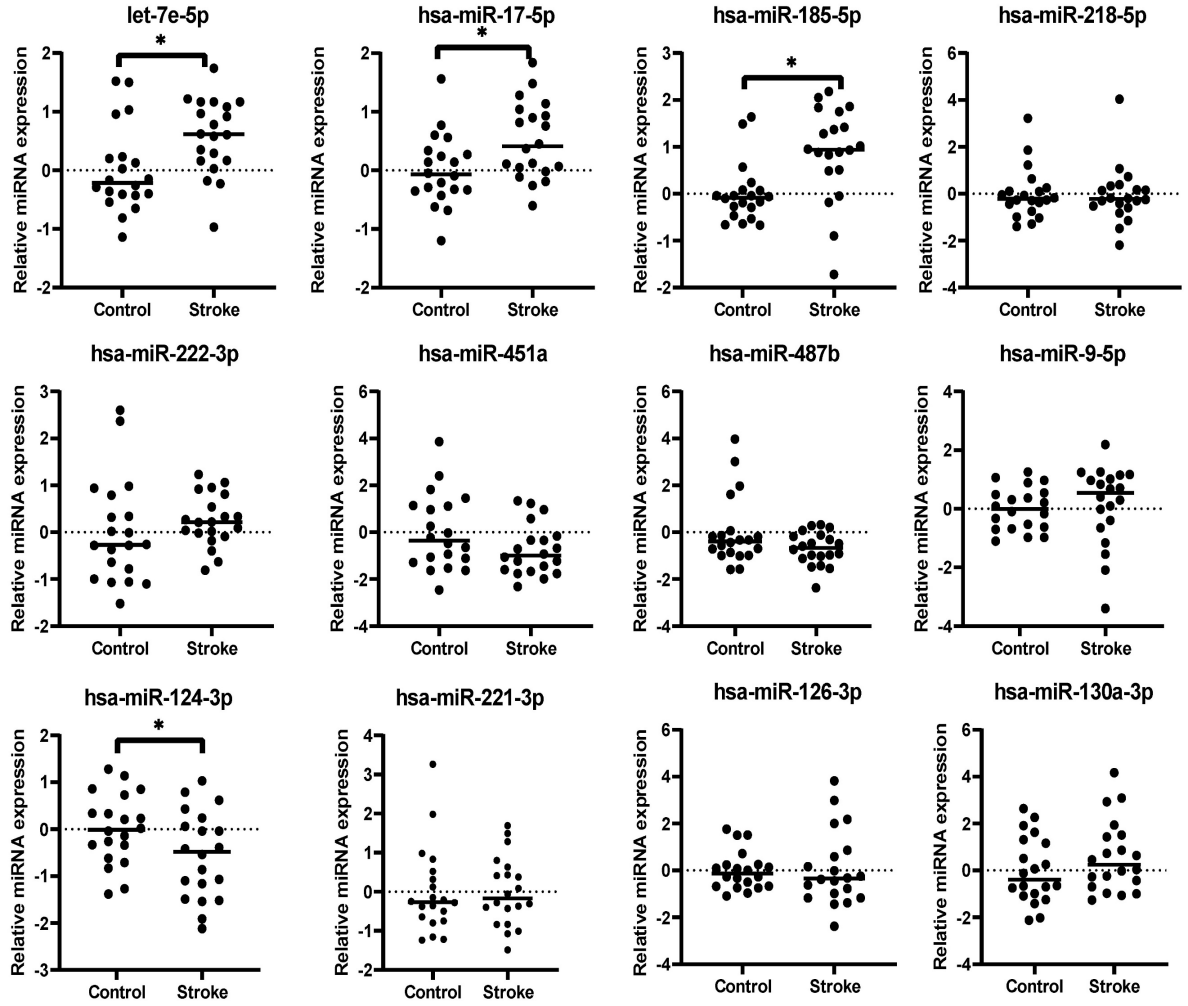
Relative expression levels of 12 miRNAs obtained from plasma from IS patients (Stroke) and healthy individuals (Control). The relative expression levels of the miRNAs are presented as ΔCt relative to the external references including cel-miR-39, 54 and 238. Fold change was calculated relative to that of healthy individuals' group. *: *p* < 0.05.

The 12 miRNAs identified from meta-analysis were subjected to qRT-PCR validation using plasma samples. Relative expression levels of each miRNA were measured in both IS (n=20) and healthy (n=20) samples (Figure 3). The expression levels of 4 miRNAs were significantly dysregulated in IS patients when compared to the healthy controls, with 3 upregulated (hsa-let-7e-5p, Fold-change[FC] = 1.50, *p* = 0.011; hsa-miR-17-5p, FC = 1.42, *p* = 0.015; hsa-miR-185-5p, FC = 1.83, *p* = 0.0019) and 1 downregulated (hsa-miR-124-3p, FC = 0.68, *p* = 0.044) (Table 3). The dysregulation states of these miRNAs were in the same directions as obtained from the results of meta-analysis, further suggesting that these 4 miRNAs could be potential biomarkers for IS patients.

### Biological significance of validated miRNAs

**Figure 4:**
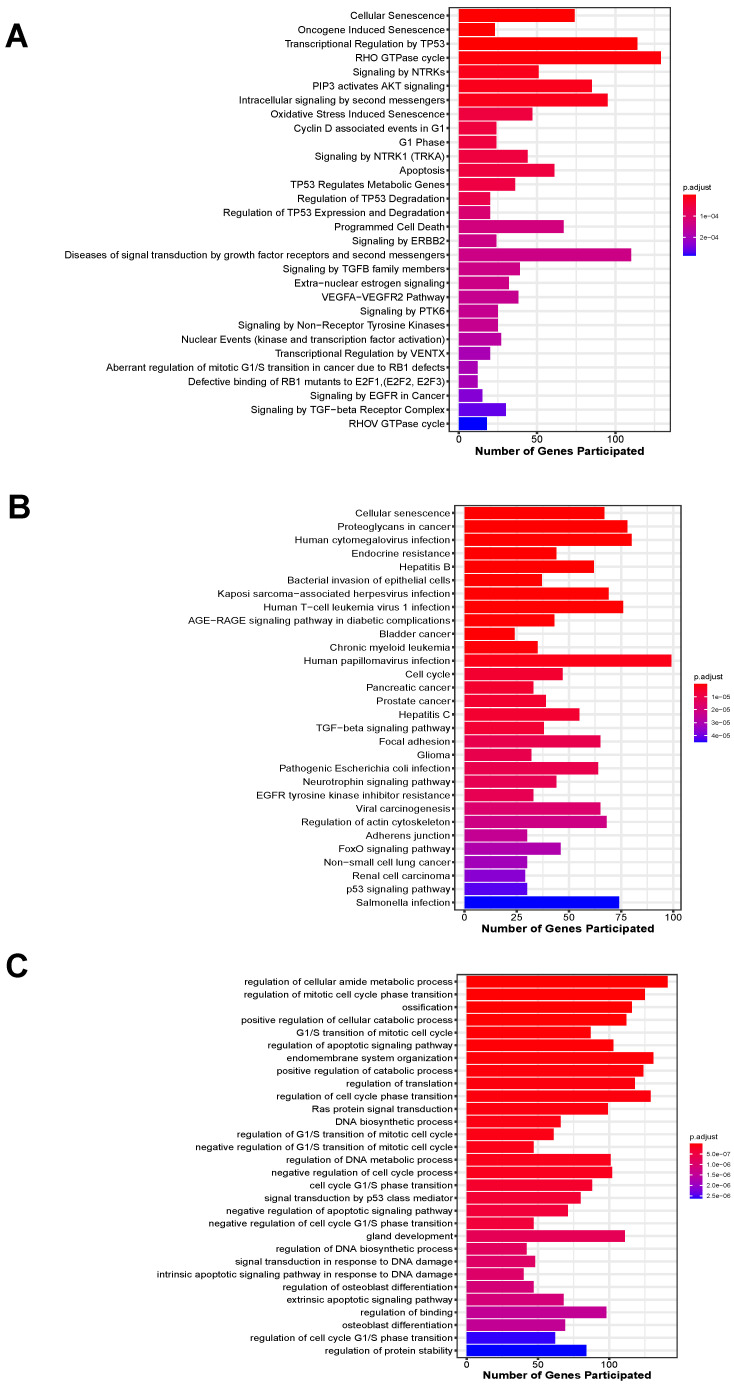
Top 30 significantly enriched pathways by target genes of the 4 miRNAs ranked by their adjusted p-value. (A) Enriched Reactome pathways; (B) Enriched KEGG pathways; (C) Enriched biological processes.

In total, 3201 unique target genes were identified from the 4 validated miRNAs (Suppl. Table S2). Pathway enrichment analysis identified 310 and 103 significantly enriched Reactome and KEGG pathways, respectively (Suppl. Table S3-S4). For enriched Reactome pathways, many of them are involved in the response to the depletion of oxygen and glucose caused by IS, including the responses to stress, pathways involved in cellular senescence, regulation of cell-cycle progression, VEGF signaling and AKT signaling (Figure 4A). Other pathways related to neuroinflammatory responses were also implicated, such as Forkhead box protein (FoxO)-mediated transcription and Toll-like receptor (TLR) cascades. These pathways are involved in apoptosis, which was also enriched, as well as mediating cell proliferation and angiogenesis to compensate for the lack of blood supply to vital organs. For KEGG-enriched pathways, similar observations were also obtained, with the addition of other cancer-associated and immune system-related pathways (Figure 4B).

Moreover, a wide range of biological processes were enriched by the target genes for the 4 miRNAs (Suppl. Table S5). Target genes of all the 4 miRNAs were implicated in the top 10 significantly enriched biological processes, including regulation of cellular metabolic and catabolic processes, ossification, cell cycle progression, apoptosis, endomembrane system organization and translation (Figure 4C).

**Figure 5:**
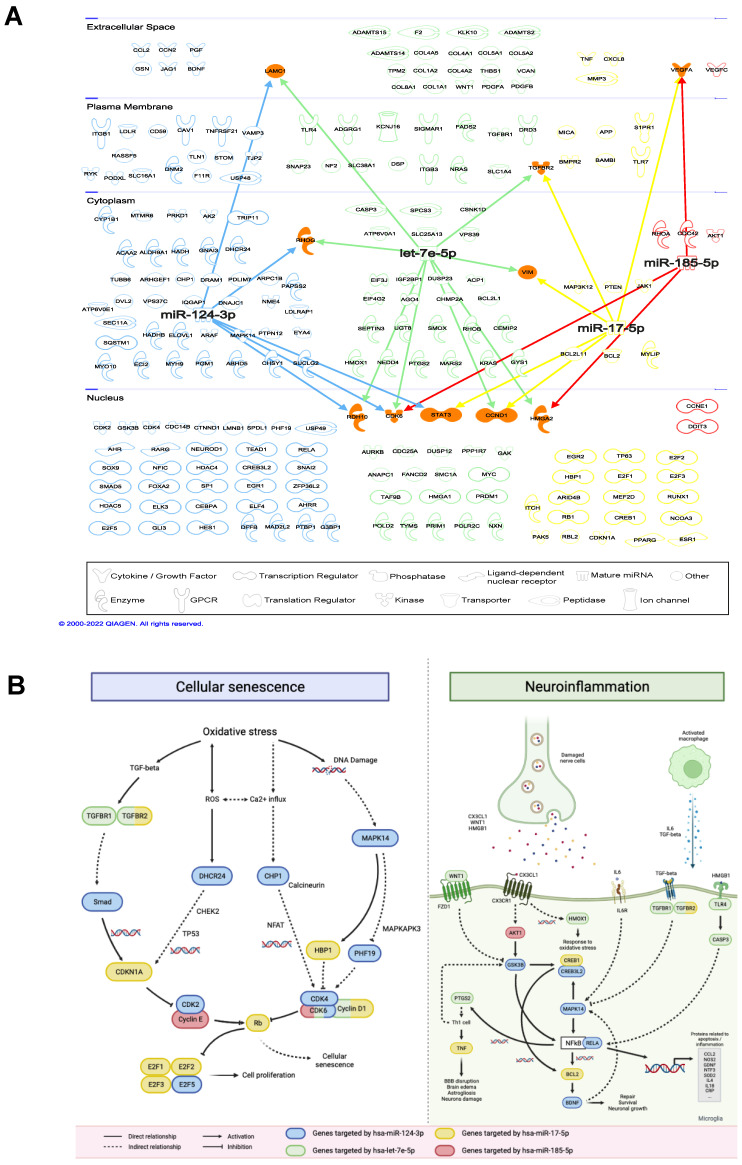
Interactions between miRNAs and target mRNAs. (A) Network visualization of 4 validated miRNAs and their target mRNAs. (B) Cellular senescence (left) and neuroinflammation (right) pathways involving miRNAs and their target mRNAs.

IPA analysis showed that 221 mRNAs known to be implicated in inflammatory response, cardiovascular or neurological diseases were targeted by the 4 miRNAs (Figure 5A, Suppl. Table S6). It was found that different mRNAs were commonly targeted by multiple miRNAs, including cardiac growth- and cell cycle-relevant genes 24, 25. For all 221 mRNAs, IS-related pathways were identified, including cellular senescence and neuroinflammation (Figure 5B). Target genes of the 4 miRNAs are involved in multiple steps in the two illustrated pathways. Genes that play important roles from activating and transducing the signaling cascades to causing cellular senescence directly are targeted by these miRNAs. In particular, *TGFBR2* is targeted by hsa-let-7e-5p and hsa-miR-17-5p that causes the transcription of *CDKN1A*, *CDK6* is targeted by hsa-let-7e-5p, hsa-miR-124-3p and hsa-miR-185-5p which inhibits the retinoblastoma protein (pRB). In neuroinflammation, multiple signaling cascades are activated by the binding of ligands released by the damaged nerve cells and macrophages to the receptors expressed on microglia. Particularly, hsa-miR-124-3p targets multiple important genes in neuroinflammation, including *GSK3B*, *CREB3L2*, *MAPK14*, *RELA* and *BDNF*. These genes interact with each other directly or indirectly to mediate the production of pro- or anti-inflammatory proteins that cause further neuronal damage or neuronal growth and repair.

### Diagnostic value of 4 validated miRNAs

**Figure 6:**
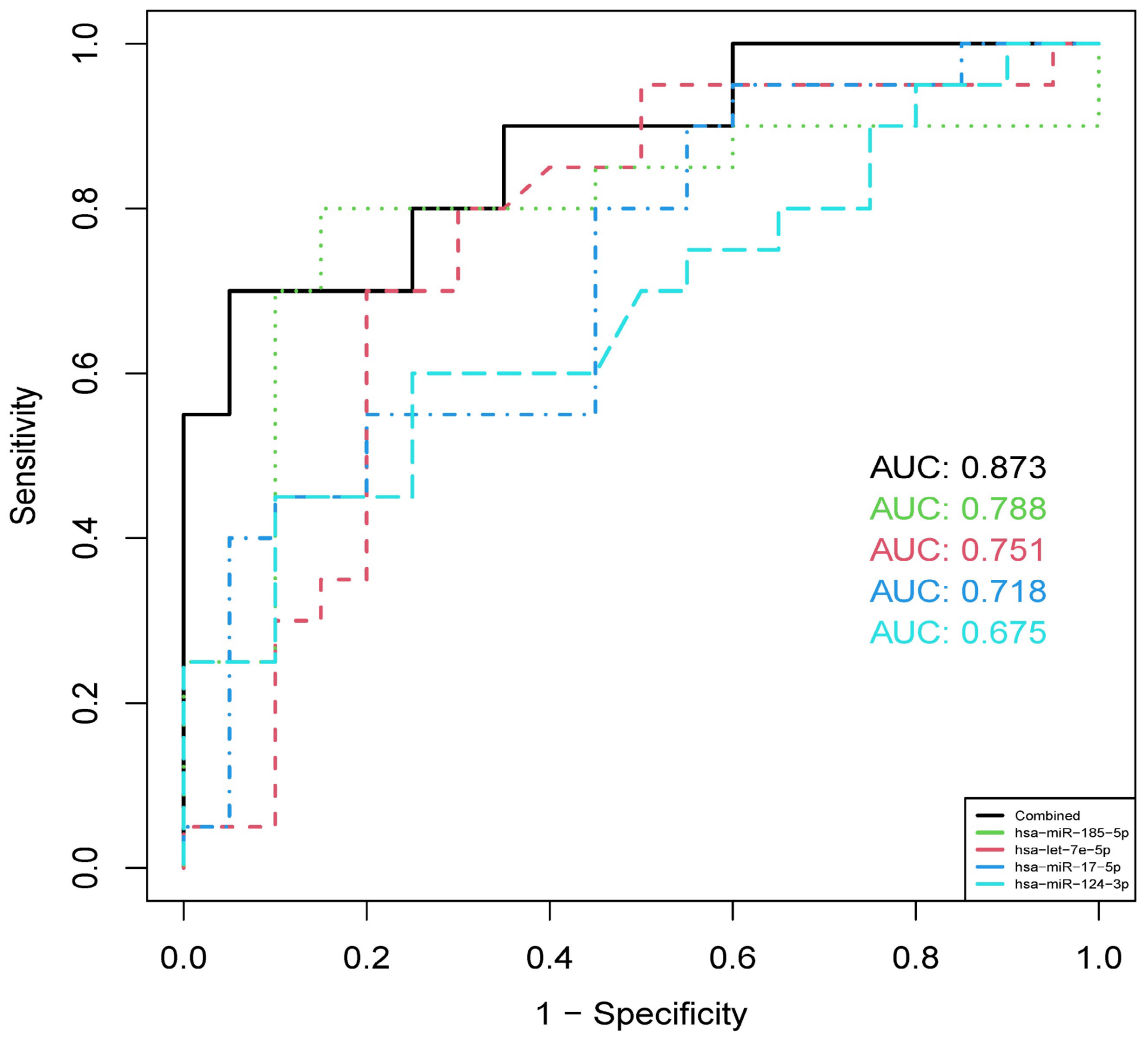
ROC curves of 4 validated miRNAs and their combined effect in distinguishing IS and healthy individuals.

To investigate the diagnostic value of identified miRNAs, cross-validation ROC curve analysis was performed on each of the 4 validated miRNAs in the plasma of all 40 samples collected from the hospital. The analysis indicated that hsa-miR-185-5p showed highest diagnostic value (AUC = 0.788), followed by hsa-let-7e-5p (AUC = 0.751), hsa-miR-17-5p (AUC = 0.718) and hsa-miR-124-3p (AUC = 0.675). When combining the 4 miRNAs, we found that the AUC was improved to 0.873 (Figure 6), suggesting the potential use of a panel of these circulating miRNAs in the diagnosis of IS.

## Discussion

The development of circulating biomarkers for IS is urgently needed to aid in predicting the risk of disease onset and early diagnosis for better clinical outcomes. miRNAs have been identified from previous studies demonstrating the potential use of these biomarkers to diagnose patients with cancers by expression profiling 26-28. In addition to diagnosis, miRNAs may also exhibit anti-tumour properties, as suggested by a study of miR-146b inhibiting cell growth of malignant glioma 29. Studies have demonstrated the therapeutic potential of miRNAs by identifying the miRNA-mRNA interactions and gene regulatory networks. For example, high-throughput sequencing of RNAs isolated by crosslinking immunoprecipitation was used to identify the miRNA-target mRNA interaction sites 30. CRISPR screening was used to identify the essential miRNA binding sites 31, while multiple *in silico* approaches could also be used to predict miRNA-target interactions 32.

Furthermore, miRNA replacement therapy and inhibitory antimiRs have also been suggested to directly target the dysregulated miRNAs as therapeutics 33. Moreover, the miRNA expression levels between disease subtypes could be distinguished, raising the potential of using miRNAs to discover personalized therapeutic targets 34. In this study, meta-analysis was performed to identify the associations between circulating miRNAs expression levels and IS. Twelve miRNAs were identified from publicly available differential expression studies as potential biomarkers, of which 4 were validated using plasma samples obtained from IS patients. Previous studies have suggested that the time of samples collection could lead to variations in the expression of circulating miRNAs 35, 36. In line with our validation results, the studies that provided the qualified miRNAs in our meta-analysis which collected the samples at 6, 48 and 72 hours did not yield significant differences in expression levels (i.e., hsa-miR-487b and hsa-miR-221-3p). This further suggests that when using circulating miRNAs as early diagnostic biomarkers, the time after stroke onset plays a key role in determining the accuracy and reliability of the diagnostic panel.

Following the events of ischemia, a series of cellular responses is activated. First, stress signals trigger neuroinflammatory responses, leading to the generation of reactive oxygen species and reactive nitrogen species which eventually causes neuronal cell death. Matrix metalloproteinase is also activated by stress signals, damaging the endothelial cells and causing dysfunction of the blood-brain barrier. Second, ion imbalance in brain cells due to reduced blood flow causes the release of glutamate and calcium ion influx. Consequently, degradative enzymes are activated, inducing dysfunction of mitochondria, cell and DNA damage, resulting in neuronal cell apoptosis 37. The target genes for the miRNAs identified by this meta-analysis are implicated in multiple pathways and biological processes that correspond to these cellular responses to IS.

Neuroinflammation is known to be associated with IS due to the release of cytokines and chemokines by the damaged neuronal cells and immune cells 38, 39. Microglia dysregulation has been associated with neuroinflammation induced by the binding of CX3CL1, IL-6, TGF-β and other cytokines 40-42. Activated glial cells then initiate intracellular signaling cascades to trigger the release of both pro- and anti-inflammatory signals 43. In this study, we identified several miRNAs in the circulation of IS patients that contribute to these mechanisms. First, binding of CX3CL1 to CX3CR1 activates the PI3K/GSK3β/NF-kβ signaling pathway which has been associated with neuroinflammation in multiple neurological disorders to regulate the release of pro- and anti-inflammatory cytokines 44. We identified hsa-miR-185-5p and hsa-miR-124-3p that target *AKT1* and *GSK3B* which both play a crucial role in this pathway. hsa-miR-124-3p also targets *RELA*, which is a subunit of the NFkB dimer that regulates the transcription of *BDNF*, where increased BDNF production promotes neuronal growth and survival 45. This pathway has also been suggested to promote the resolution of neuroinflammation and increase tissue repair 46. Our finding agrees with previous studies that down-regulation of hsa-miR-124-3p was observed in an Alzheimer's Disease (AD) model, contributing to neuroprotection via the regulation of PI3K/Akt/GSK3β pathway in neurological diseases to reduce the release of pro-inflammatory cytokines 47. hsa-miR-185-5p was also reported to participate in the neuro-protective axes in response to brain injury and AD 48, 49. Second, hsa-let-7e-5p targets *WNT1* and multiple receptors that initiate the signaling cascades in neuroinflammation including TGFBR1/2 and TLR4. hsa-let-7e-5p also targets *HMOX1* that is responsible for reducing oxidative stress and tissue damage 50, 51. hsa-miR-17-5p targets TNF-α which is a pro-inflammatory cytokine known to be involved in neuroinflammation 52. Similar to hsa-miR-124-3p and hsa-miR-185-5p, hsa-miR-17-5p was upregulated in both IS and AD patients 53. Other studies suggested the relationship between miR-17-5p, SMAD7, and TNF-α, where overexpression of miR-17-5p negatively regulates the expression of SMAD7 that increases the release of TNF-α and other cytokines 53, 54. Therefore, up-regulation of miR-17-5p in the circulation may be a potential biomarker to reflect the neuroinflammatory response post-IS.

Cellular senescence plays an important role in the response to IS. As a response to the stressful stimuli such as oxidative stress and DNA damage, cellular senescence leads to impaired cell replication. The association between cellular senescence and IS was recently suggested by a study observing the cellular senescence-associated secretory phenotype in mice and human with IS 55. The relationship between the shortening of cell-cycle, especially G1-phase, and IS patients was also suggested as a result of increased stroke-induced neurogenesis. Our results are in agreement with previous studies. hsa-miR-17-5p which targets *CDKN1A* and pRB was identified in this study. It has been well known that both *CDKN1A* and pRB play a crucial role in inhibiting cell cycle progression and inducing senescence 56, 57. Evidence has shown that another hsa-miR-17-5p's target, *HBP1*, contributes to premature cellular senescence by either activating p16 or repressing DNMT1, that indirectly inhibits the transcription of cyclin-D1 58. Cyclin-D1, encoded by *CCND1*, is targeted by both hsa-let-7e-5p and hsa-miR-17-5p. This protein is overexpressed in senescent cells due to its action of preventing the cells from entering S phase 59. Agreeing with previous studies, the let-7 family members are highly implicated in cancers by acting as tumor suppressors and mediating cell cycle dynamics by regulating different checkpoints 60, 61.

Apart from the pathways regulating cell cycle progression, the VEGF-associated pathways implicated by the target genes of miRNAs regulating angiogenesis correspond to the literatures suggesting as one of the mainly affected pathways in post-IS patients 62. Neurotrophin signaling pathway is responsible for the repair and regeneration of neuronal cells after ischemic or traumatic brain injury 63. Moreover, FoxO signaling pathway was also implicated. Its roles in IS have also been suggested, including the induction of apoptosis, inflammation and affecting the blood-brain barrier 64.

Therefore, our results correlate with existing literature demonstrating the associations between specific pathways and IS. Our study identified 4 reliable associations between the miRNAs and IS, highlighting the potential of targeting them or their target genes for developing diagnostic or therapeutic tools.

## Supplementary Material

Supplementary tables.

## Figures and Tables

**Table 1:** Characteristics of the included studies and the involved miRNAs.

PMID	Author	Year	Country	miRNAs	Dysregulation state	Sample size		Specimen	Technique
						Stroke	Healthy		
19888324	Tan, K. S.	2009	Singapore	hsa-miR-126-3phsa-miR-144-3phsa-miR-16-5phsa-miR-21-5phsa-miR-223-3phsa-miR-320a-3p	DownUpUpUpUpUp	19	5	Whole blood	microarray, qRT-PCR
21622133	Zeng, L.	2011	China	hsa-miR-210-3p	Down	112	60	Whole blood	qRT-PCR
24237608	Long, G.	2013	China	hsa-miR-126-3phsa-miR-30a-5p	DownDown	38	50	Plasma	qRT-PCR
24911610	Jickling, G. C.	2014	USA	hsa-let-7i-5phsa-miR-122-5phsa-miR-148a-3phsa-miR-19a-3phsa-miR-320dhsa-miR-363-3phsa-miR-4429hsa-miR-487b-3p	DownDownDownDownDownUpDownUp	24	24	PBMCs	microarray, qRT-PCR
25257664	Liu, Y.	2015	China	hsa-miR-124-3phsa-miR-9-5p	DownDown	31	11	Serum	qRT-PCR
25287657	Wang, W.	2014	China	hsa-miR-106b-5phsa-miR-320dhsa-miR-320ehsa-miR-4306	UpDownDownUp	136	116	Plasma	microarray, qRT-PCR
25410304	Li, P.	2015	China	hsa-miR-1246hsa-miR-1299hsa-miR-1913hsa-miR-224-3phsa-miR-3149hsa-miR-32-3phsa-miR-377-5phsa-miR-423-5phsa-miR-451ahsa-miR-4739hsa-miR-518bhsa-miR-532-5p	UpUpDownDownUpUpDownUpUpUpDownDown	117	82	Serum	microarray, qRT-PCR
26044809	Li, S.	2015	China	hsa-miR-146a-5phsa-miR-185-5p	Down Down	60	30	Whole blood	microarray, qRT-PCR
26096228	Jia, L.	2015	China	hsa-miR-145-5phsa-miR-221-3phsa-miR-23a-3p	UpDownDown	146	96	Serum	qRT-PCR
26459744	Zeng, Y.	2015	China	hsa-miR-124-3phsa-miR-218-5phsa-miR-22-3phsa-miR-23a-3phsa-miR-30a-5phsa-miR-33a-5phsa-miR-330-3phsa-miR-9-5p	DownDownUpUpDownDownDownDown	10	10	Serum	qRT-PCR
26885038	Wu, J.	2015	China	hsa-miR-15a-5phsa-miR-16-5phsa-miR-17-5p	UpUpUp	106	120	Serum	qRT-PCR
27545688	Liang, T.	2016	China	hsa-miR-34a-5p	Up	102	97	Plasma	qRT-PCR
27776139	Huang, S.	2016	China	hsa-let-7e-5p	Up	302	302	Whole blood	qRT-PCR
28111007	Wang, Y.	2017	China	hsa-miR-221-3phsa-miR-382-5p	DownDown	68	39	Serum	qRT-PCR
28168424	Bam, M.	2018	USA	hsa-miR-130a-3phsa-miR-320ahsa-miR-376c-3phsa-miR-432-5phsa-miR-4656hsa-miR-487hsa-miR-503-5phsa-miR-874-3p	UpUpUpUpUpUpUpDown	19	20	PBMCs	microarray, qRT-PCR
28875333	Jin, F.	2017	China	hsa-miR-126-3phsa-miR-130a-3phsa-miR-185-5phsa-miR-218-5phsa-miR-222-3phsa-miR-378a-5p	DownDownUpUpUpDown	106	110	Plasma	qRT-PCR
29402769	Chen, Z.	2018	China	hsa-miR-146b-5p	Up	128	102	Serum	qRT-PCR
29701837	Vijayan, M.	2018	USA	hsa-miR-122-5phsa-miR-211-5phsa-miR-22-3phsa-miR-23a-3phsa-miR-30d-5p	UpUpDownDownDown	34	11	Serum	Illumina deep sequencing, qRT-PCR
30030634	Jin, F.	2018	China	hsa-miR-126-3phsa-miR-130a-3phsa-miR-185-5phsa-miR-219-5phsa-miR-222-3p	DownDownUpUpUp	148	148	Plasma	qRT-PCR
30112629	Yoo, H.	2019	Korea	hsa-let-7e-5phsa-miR-1229-3phsa-miR-1238-5phsa-miR-1270hsa-miR-1294hsa-miR-1301-3phsa-miR-140-5phsa-miR-142-3phsa-miR-144-3phsa-miR-186-5phsa-miR-18b-5phsa-miR-19a-3phsa-miR-301a-3phsa-miR-32-5phsa-miR-335-5phsa-miR-340-5phsa-miR-362-3phsa-miR-505-5phsa-miR-517b-3phsa-miR-544ahsa-miR-579-3phsa-miR-628-5phsa-miR-660-5phsa-miR-664a-5phsa-miR-877-5p	UpUpUpUpUpUpDownDownDownDownDownDownDownDownDownDownDownUpDownUpDownUpDownUpUp	10	11	Whole blood	microarray, TaqMan miRNA assay
30617992	van Kralingen, J. C.	2019	UK	hsa-miR-17-5phsa-miR-20b-5phsa-miR-27b-3phsa-miR-93-5p	UpUpUpUp	139	34	Serum	microarray, qRT-PCR
30678250	Giordano, M.	2019	Italy	hsa-miR-195-5phsa-miR-451a	UpUp	18	20	Serum	qRT-PCR
30899379	Geng, W.	2019	China	hsa-miR-126-3p	Down	13	17	Plasma	qRT-PCR
31496785	Kotb, H. G.	2019	Egypt	hsa-miR-146a-5p	Down	44	22	Serum	qRT-PCR
31935511	Li, L.	2020	China	hsa-miR-1275	Down	279	279	Whole blood	microarray, qRT-PCR
32406219	Li, S.	2020	China	hsa-miR-128-3p	Up	80	60	Serum	qRT-PCR
35018114	Guo, C.	2022	China	hsa-miR-185-5phsa-miR-424-5p	UpUp	142	50	Serum	qRT-PCR
35328807	Aldous, E. K.	2022	Qatar	hsa-miR-451ahsa-miR-574-5phsa-miR-4446-3phsa-miR-142-3phsa-miR-6721-5phsa-miR-676-3phsa-miR-379-5phsa-miR-485-3phsa-miR-411-5phsa-miR-149-5p	UpDownDownDownDownDownDownDownDownDown	47	96	Serum	RNA-sequencing
35562921	Eyileten, C.	2022	Poland	hsa-miR-19a-3phsa-let-7f-5p	UpDown	28	35	Plasma	qRT-PCR

**Table 2:** Meta-analysis results for qualified miRNAs.

miRNAs	Study	Specimen	τ^2	*I^2*	Weight	P-value	LogOR	95% CI
**hsa-let-7e-5p**	Huang, S., 2016Yoo, H., 2019	Whole blood	17.89	81.37%	50.20%49.80%	4.13E-03	9.51	[3.01, 16.01]
**hsa-miR-124-3p**	Liu, Y., 2015Zeng, Y., 2015	Serum	0	0.00%	50.43%49.57%	3.46E-06	-6.69	[-9.51, -3.86]
**hsa-miR-126-3p**	Long, G., 2013Jin, F., 2017Jin, F., 2018Geng, W., 2019	Plasma	0.09	2.14%	25.13%25.17%25.20%24.50%	1.75E-22	-9.92	[-11.91, -7.93]
**hsa-miR-130a-3p**	Jin, F., 2017Jin, F., 2018	Plasma	0	0.00%	49.97%50.03%	5.51E-15	-11.07	[-13.85, -8.30]
**hsa-miR-144-3p**	Tan, K. S., 2009Yoo, H., 2019	Whole blood	57.53	94.20%	49.44%50.56%	9.31E-01	0.47	[-10.36, 11.30]
**hsa-miR-17-5p**	Wu, J., 2015van Kralinge, J. C., 2019	Serum	0	0.00%	50.11%49.89%	2.90E-13	10.36	[7.58, 13.14]
**hsa-miR-185-5p**	Jin, F., 2017Jin, F., 2018	Plasma	0	0.00%	49.97%50.03%	5.51E-15	11.07	[8.30, 13.85]
**hsa-miR-218-5p**	Jin, F., 2017Jin, F., 2018	Plasma	0	0.00%	49.97%50.03%	5.51E-15	11.07	[8.30, 13.85]
**hsa-miR-22-3p**	Zeng, Y., 2015Vijayan, M., 2018	Serum	86.41	95.41%	49.98%50.02%	9.24E-01	-0.64	[-13.83, 12.55]
**hsa-miR-221-3p**	Jia, L., 2015Wang, Y., 2017	Serum	0	0.00%	50.14%49.86%	1.01E-12	-10.12	[-12.90, -7.34]
**hsa-miR-222-3p**	Jin, F., 2017Jin, F., 2018	Plasma	0	0.00%	49.97%50.03%	5.51E-15	11.07	[8.30, 13.85]
**hsa-miR-23a-3p**	Jia, L., 2015Zeng, Y., 2015Vijayan, M., 2018	Serum	76.5	94.90%	33.37%33.30%33.33%	4.31E-01	-4.08	[-14.24, 6.08]
**hsa-miR-451a**	Li, P., 2015Giordano, M., 2019	Serum	1.19	22.67%	50.39%49.61%	3.24E-08	8.96	[5.78, 12.13]
**hsa-miR-487b-3p**	Jickling, G. C., 2014Bam, M., 2018	PBMCs	0	0.00%	50.11%49.89%	1.15E-07	7.58	[4.78, 10.38]
**hsa-miR-9-5p**	Liu, Y., 2015Zeng, Y., 2015	Serum	0	0.00%	50.43%49.57%	3.46E-06	6.69	[3.86, 9.51]

**Table 3:** Mean relative expression levels of 12 miRNAs in plasma of IS patients (n=20) and healthy individuals (n=20).

miRNA	Stroke	Healthy	delta delta CT	Fold-change	p-value
hsa-let-7e-5p	0.582 ± 0.143	-5e-04 ± 0.164	0.58	1.50	0.0108
hsa-miR-17-5p	0.509 ± 0.147	0.0025 ± 0.135	0.51	1.42	0.0153
hsa-miR-185	0.87 ± 0.221	-0.004 ± 0.139	0.87	1.83	0.0019
hsa-miR-218	-0.0685 ± 0.273	0.002 ± 0.244	-0.07	0.95	0.8484
hsa-miR-222	0.244 ± 0.123	-5e-04 ± 0.247	0.24	1.18	0.3809
has-miR-451a	-0.756 ± 0.243	-0.0025 ± 0.359	-0.76	0.59	0.0897
hsa-miR-487b	-0.693 ± 0.157	0.001 ± 0.332	-0.69	0.62	0.0664
hsa-miR-9-5p	0.138 ± 0.301	-5e-04 ± 0.163	0.14	1.10	0.6881
hsa-miR-124-3p	-0.552 ± 0.207	0.0035 ± 0.168	-0.55	0.68	0.0441
hsa-miR-221-3p	0.0055 ± 0.197	0.0035 ± 0.248	0.01	1.00	0.995
hsa-miR-126-3p	0.064 ± 0.356	0.0025 ± 0.185	0.06	1.05	0.8789
hsa-miR-130a-3p	0.592 ± 0.34	-0.001 ± 0.316	0.59	1.51	0.2093

**Table 4:** Biological processes enriched by target genes of all the 4 miRNAs. FDR; False discovery rate.

GO term	Biological Process	FDR
GO:0034248	Regulation of cellular amide metabolic process	7.75E-10
GO:1901990	Regulation of mitotic cell cycle phase transition	1.16E-08
GO:0001503	Ossification	1.50E-08
GO:0031331	Positive regulation of cellular catabolic process	1.50E-08
GO:0000082	G1/S transition of mitotic cell cycle	2.47E-08
GO:2001233	Regulation of apoptotic signaling pathway	2.65E-08
GO:0010256	Endomembrane system organization	3.49E-08
GO:0009896	Positive regulation of catabolic process	3.86E-08
GO:0006417	Regulation of translation	5.73E-08
GO:1901987	Regulation of cell cycle phase transition	5.73E-08

## Abbreviations

IS: ischemic stroke
miRNAs: microRNAs
PBMC: peripheral blood mononuclear cells
CSF: cerebrospinal fluid
OR: odds ratios
KEGG: Kyoto Encyclopedia of Genes and Genomes
GO: Gene Ontology
FDR: False Discovery Rate
IPA: Ingenuity Pathway Analysis
ROC: Receiver operating characteristics
